# Evaluation of nucleotide MALDI-TOF-MS for the identification of *Mycobacterium* species

**DOI:** 10.3389/fcimb.2024.1335104

**Published:** 2024-02-06

**Authors:** Yelei Zhu, Zhengwei Liu, Lina Peng, Bin Liu, Kunyang Wu, Mingwu Zhang, Xiaomeng Wang, Junhang Pan

**Affiliations:** ^1^ Department of Tuberculosis Control and Prevention, Zhejiang Provincial Center for Disease Control and Prevention, Hangzhou, China; ^2^ Department of Service and Support, Agena Bioscience, Shanghai, China

**Keywords:** nucleotide MALDI-TOF-MS, species identification, NTM, MTBC, mixed infection

## Abstract

**Background:**

The accurate identification of the *Mycobacterium* tuberculosis complex (MTBC) and different nontuberculous mycobacteria (NTM) species is crucial for the timely diagnosis of NTM infections and for reducing poor prognoses. Nucleotide matrix-assisted laser desorption/ionization time-of-flight mass spectrometry (MALDI-TOF-MS) has been extensively used for microbial identification with high accuracy and throughput. However, its efficacy for *Mycobacterium* species identification has been less studied. The objective of this study was to evaluate the performance of nucleotide MALDI-TOF-MS for *Mycobacterium* species identification.

**Methods:**

A total of 933 clinical *Mycobacterium* isolates were preliminarily identified as NTM by the MPB64 test. These isolates were identified by nucleotide MALDI-TOF-MS and Sanger sequencing. The performance of nucleotide MALDI-TOF MS for identifying various *Mycobacterium* species was analyzed based on Sanger sequencing as the gold standard.

**Results:**

The total correct detection rate of all 933 clinical *Mycobacterium* isolates using nucleotide MALDI-TOF-MS was 91.64% (855/933), and mixed infections were detected in 18.65% (174/933) of the samples. The correct detection rates for *Mycobacterium intracellulare*, *Mycobacterium abscessus*, *Mycobacterium kansasii*, *Mycobacterium avium*, MTBC, *Mycobacterium gordonae*, and *Mycobacterium massiliense* were 99.32% (585/589), 100% (86/86), 98.46% (64/65), 94.59% (35/37), 100.00% (34/34), 95.65% (22/23), and 100% (19/19), respectively. For the identification of the MTBC, *M. intracellulare*, *M. abscessus*, *M. kansasii*, *M. avium*, *M. gordonae*, and *M. massiliense*, nucleotide MALDI-TOF-MS and Sanger sequencing results were in good agreement (*k* > 0.7).

**Conclusion:**

In conclusion, nucleotide MALDI-TOF-MS is a promising approach for identifying MTBC and the most common clinical NTM species.

## Introduction

Nontuberculous mycobacteria (NTM) comprise a large group of acid-fast mycobacteria, apart from the *Mycobacterium tuberculosis* complex (MTBC) and *Mycobacterium leprae*. NTM are opportunistic pathogens that frequently occur in natural settings, including soil, air, and water. There are approximately 200 NTM species or subspecies, owing to the large number of newly discovered species in recent decades (Larsson et al., 2017; Forbes et al., 2018), and the incidence of diseases caused by NTM has gradually increased worldwide (Brode et al., 2014). NTM is more common than tuberculosis (TB) in many developed countries (Baldwin et al., 2019), and the rate of NTM isolation in China is increasing (Wang et al., 2014). In Zhejiang Province, a coastal province in southeast China, the increased isolation rate of NTM and the diversity of NTM species are pronounced (Yu et al., 2016). Human NTM infections primarily lead to lung, skin, bone, joint, and superficial lymph node disease. Among these, NTM pulmonary disease is the most prevalent and has attracted increasing attention as an emerging public health challenge (Hoefsloot et al., 2013).

However, most NTM strains are resistant to the commonly administered anti-TB agents. Moreover, mixed NTM–MTB infections can be misdiagnosed as drug-resistant TB (Huang et al., 2022). This leads to a delayed diagnosis and poor treatment outcomes. Further, the increase in NTM-associated mortality in the aging population requires more attention (Park et al., 2019). In addition, drug resistance spectra, regimens, and therapeutic efficacies vary widely among different NTM species (Kang and Koh, 2016). Therefore, the accurate identification of the MTBC and different NTM species is crucial for timely NTM diagnoses and improving poor prognoses.

Many molecular techniques are available for rapid *Mycobacterium* species identification, including GenoType *Mycobacterium* CM/AS (Hain Lifescience GmbH, Nehren, Germany), DNA microarray chip (Capital Bio Technology Inc., Beijing, China), and the MeltPro Myco assay (Zeesan Biotech, Xiamen, China). However, these methods are expensive, and their scope for species detection is limited. Differentiating among close species within some complex groups, such as *Mycobacterium intracellulare*, *Mycobacterium chimaera*, and *Mycobacterium marseillense*, within the *Mycobacterium avium* complex (MAC), and *Mycobacterium abscessus* and *Mycobacterium massiliense*, within the *M. abscessus* complex (MABC), is challenging (Mäkinen et al., 2006; Liu et al., 2012; Xu et al., 2019).

Sanger sequencing is considered the gold standard for identifying a wide range of *Mycobacterium* species, as it can identify *Mycobacterium* at the species or subspecies levels (Kim and Shin, 2018). However, its time-consuming and technically complex nature, as well as the limitations associated with the detection of mixed infections, make it unsuitable for use in medical institutions (Forbes et al., 2018; Liang et al., 2020). Matrix-assisted laser desorption/ionization time-of-flight mass spectrometry (MALDI-TOF MS) is a valuable technique for biomolecular analyses (Meyer and Ueland, 2011). When combined with multiplex PCR technology, it can be broadly applied to several aspects of genomic research, including single-nucleotide polymorphism, mutation, DNA methylation analyses, typing, and microorganism detection (Vogel et al., 2009), as well as virus and microbe detection. Numerous studies have demonstrated the efficacy of nucleotide MALDI-TOF-MS for identifying pathogenic microorganisms and analyzing their resistance patterns (Bouakaze et al., 2011; Zhang et al., 2018; Wu et al., 2022). Furthermore, compared to Sanger sequencing, it is more specific and sensitive and has detection limits comparable to those of other modern technologies, such as next-generation sequencing (Trembizki et al., 2014; Kriegsmann et al., 2015).

However, few studies on the identification of *Mycobacterium* species based on nucleotide MALDI-TOF-MS technology have been published. According to Li et al. (Li et al., 2022), this technique can be used to identify mycobacteria with adequate sensitivity and specificity and a low limit of detection (LOD). However, because of resource constraints, in that study, only its ability to distinguish tuberculosis from NTM infection was evaluated, and it did not include sufficient samples containing NTM (Li et al., 2022). In this study, the performance of nucleotide MALDI-TOF-MS for *Mycobacterium species* identification was assessed.

## Materials and methods

### Clinical isolates

In total, 933 clinical *Mycobacterium* isolates were collected from 35 designated TB hospitals in 11 cities in Zhejiang Province, China, between January 2020 and October 2021. All isolates were cultivated using a BACTEC MGIT 960 Mycobacterial Detection System (Becton Dickinson, Baltimore, MD, USA) and were preliminarily identified as NTM using the MPB64 test (Hangzhou Genesis Biodetection & Biocontrol Co., Ltd., Hangzhou, China). All strains were shipped to the Zhejiang Provincial Center for Disease Control and Prevention and re-cultivated in Löwenstein–Jensen (L–J) medium.

### DNA extraction

A loopful of *Mycobacterium* culture was scraped from the L–J medium and suspended in 300 μL of TE buffer (pH8.0; 10 mM Tris-HCl and 1 mM ethylenediaminetetraacetic acid). The suspension was lysed at 95 °C for 30 min and centrifuged at 15,000 × *g* for 1 min. The DNA-containing supernatant was then aspirated and stored at −20 °C.

### Nucleotide MALDI-TOF-MS detection

Nucleotide MALDI-TOF-MS assays were performed using the MassARRAY^®^ System (Agena Bioscience, San Diego, CA, USA). The identification panel for *Mycobacterium* species is of commercial synthesis from Agena Bioscience (Shanghai, China). The panel was designed based on the isolation of species from Chinese populations (Liu et al., 2021; Tan et al., 2021). Most clinically common species were included in well 1, while clinically uncommon species were mainly contained in well 2 (**Table 1**).

**Table 1:** The identification panel for *Mycobacterium* species.

Well 1	Well 2
*M. tuberculosis* complex	*M. terrae*
*M. abscessus*	*M. gastri*
*M. avium*	*M. massiliense*
*M. kansasii*	*M. septicum*
*M. intracellulare*	*M. peregrinum*
*M. scrofulaceum*	*M. mucogenicum*
*M. asiaticum*	*M. gilvum*
*M. celatum*	*M. triviale*
*M. fortuitum*	*M. phlei*
*M. xenopi*	*M. marinum*
*M. shimoidei*	*M. gordonae*
*M. chelonae*	*M. chimaera*
*M. szulgai*	*M. simiae*
*M. haemophilum*	*M. genavense*
*M. malmoense*	
*M. smegmatis*	
*M. ulcerans*	

### PCR

The DNA samples were subjected to multiplexed PCR amplification using the iPLEX pro kit (Agena Bioscience, San Diego, CA, USA), following the instructions provided by the manufacturer, with the following modifications: a 10 μL reaction volume for each sample, which contained a mixture of 1× PCR buffer, 2 mM MgCl_2_, 500 μM deoxynucleoside triphosphates (dNTPs), 0.1 μM of each PCR primer, 0.5 U of PCR enzyme, and 6.5 μL of the DNA template (10 ng/μL). An initial denaturation stage for 2 min at 95 °C was followed by 45 cycles of denaturation for 30 s at 95°C, annealing for 30 s at 56°C, extension for 1 min at 72°C, and finally a final extension for 5 min at 72°C.

### Shrimp alkaline phosphatase reaction

After PCR amplification, 0.5 U of the SAP enzyme was added to the PCR products to neutralize the unincorporated dNTPs. After 40 min of incubation at 37 °C, the samples were heated for 5 min at 85 °C to inactivate the enzyme.

### Extension reaction

Four microliters of the iPLEX pro-extension reaction cocktail were added to the PCR products that were previously treated with SAP, along with 0.222× iPLEX pro buffer, 1× iPLEX termination mix, 1× iPLEX enzyme, and an extension primer mix. The final reaction was conducted in a total volume of 18 μL. The sample was denatured at 94°C, strands were annealed at 52°C for 5 s and extended at 80°C for 5 s, following a 200-short-cycle program as recommended by the manufacturer. The information on the extension reaction designed for *Mycobacterium* identification is shown in **Supplementary Table 1**.

### Sample desalting

The iPLEX reaction products were desalted to optimize their spectral signals via mass spectrometry. The samples were diluted with 9 μL of water (deionized water). Subsequently, desalting was performed on the CPM module by adding 6 mg of resin to each reaction well, and the samples were mixed automatically during the desalting process.

### Mass spectrometry analysis

The MassARRAY^®^ Nanodispenser was used to spot PCR products onto the chip, which was then placed into the MassARRAY^®^ Analyzer for detection and analysis. The MassARRAY^®^ Typer was used to gather mass spectrometry data, and a self-developed bioinformatics pipeline built on embedded Perl 5 was used to analyze the data.

### Sanger sequencing

Sanger sequencing was performed to identify *Mycobacterium* species based on the sequencing of the 16S-23S rRNA gene internal transcribed spacer (Mohamed et al., 2005), *rpoB*, and *hsp65* using previously published primers and methods (Liu et al., 2016). Sanger sequencing was performed in both directions. The Basic Local Alignment Search Tool (BLAST) was used to compare the amplified sequences with the NCBI GenBank database (https://blast.ncbi.nlm.nih.gov), as previously described (Liu et al., 2016). The results were regarded as correct if the identification based on nucleotide MALDI-TOF-MS was consistent with Sanger sequencing. For mixed-infection isolates, the results were considered correct if either of the mixed-infection species was consistent with the Sanger sequencing results.

### LOD of nucleotide MALDI-TOF-MS

All targeted DNA fragments for each NTM species were synthesized and cloned into the plasmid pUC57 (Nanjing GenScript Biotech Co., Ltd., Nanjing, China) as standard samples. Absolute qPCR was performed to quantify the concentration of each DNA standard, using primers targeting a universal tag cloned into the plasmid. Furthermore, each standard was diluted to 5 copies/μL, 15 copies/μL, 50 copies/μL, and 150 copies/μL as the LOD references. Each reference sample and negative reference (deionized water) were tested three times, and the LOD was the lowest concentration that could be detected.

### Statistical analysis

The sensitivity, specificity, positive predictive value (PPV), negative predictive value (NPV), and accuracy of nucleotide MALDI-TOF MS for identifying single-infection isolates were calculated based on Sanger sequencing as the gold standard. The agreement between nucleotide MALDI-TOF-MS and Sanger sequencing was quantified using kappa statistics. All data were analyzed using the statistical software R (version 4.1.1, R Foundation for Statistical Computing, Vienna, Austria, 2021). The 95% confidence intervals are also presented.

## Results

### LOD of nucleotide MALDI-TOF-MS for *Mycobacterium* species identification

The LOD of nucleotide MALDI-TOF-MS for various *Mycobacterium* species was the lowest concentration detectable in all three replicate assays. Most targeted species could be detected at 5 copies/μL or 15 copies/μL, except for the *Mycobacterium septicum* assay, where the LOD was 50 copies/μL. Non-specific amplification caused by interactions between multiple PCR primers resulted in detection peaks at some molecular mass positions in the negative control. The data on the LOD for *Mycobacterium* species identification are shown in **Supplementary Table 2**.

### Distribution of the 933 *Mycobacterium* isolates based on Sanger sequencing

In total, 933 clinical *Mycobacterium* isolates were categorized into 17 *Mycobacterium* species based on Sanger sequencing results (**Table 2**). Among them, the six most dominant species were *M. intracellulare* (63.13%, 589/933), *M. abscessus* (9.22%, 86/933), *Mycobacterium kansasii* (6.97%, 65/933), *M. avium* (3.97%, 37/933), MTBC (3.64%, 34/933), and *M. marseillense* (3.64%, 34/933). Other species accounted for 9.43% of the total, including 23 *Mycobacterium gordonae*, 23 *Mycobacterium colombiense*, 19 *M. massiliense*, seven *Mycobacterium fortuitum*, six *Mycobacterium lentiflavum*, three *Mycobacterium parascrofulaceum*, and three *Mycobacterium interjectum* isolates.

**Table 2:** Identification results of 933 isolates based on nucleotide MALDI-TOF-MS and Sanger sequencing.

Sanger sequencing results	Total	Number (n, %) of isolates identified by nucleotide MALDI-TOF-MS			
		Correct results	Single infection	Mixed infection	Misidentification results
*M. intracellulare*	589	585(99.32%)	496	89	4(0.68%)
*M. abscessus*	86	86(100%)	69	17	0(0.00%)
*M. kansasii*	65	64(98.46%)	50	14	1(1.54%)
*M. avium*	37	35(94.59%)	18	17	2(5.41%)
*M. tuberculosis* complex	34	34(100%)	15	19	0(0.00%)
M. marseillense*	34	0(0.00%)	0	0	34(100%)
*M. gordonae*	23	22(95.65%)	11	11	1(4.35%)
M. colombiense*	23	0(0.00%)	0	0	23(100%)
*M. massiliense*	19	19(100.00%)	14	5	0(0.00%)
*M. fortuitum*	7	7(100.00%)	6	1	0(0.00%)
M. lentiflavum*	6	0(0.00%)	0	0	6(100%)
M. parascrofulaceum*	3	0(0.00%)	0	0	3(100%)
M. interjectum*	3	0(0.00%)	0	0	3(100%)
*M. chelonae*	1	1(100.00%)	0	1	0(0.00%)
*M. xenopi*	1	1(100.00%)	1	0	0(0.00%)
*M. scrofulaceum*	1	0(0.00%)	0	0	1(100%)
*M. peregrinum*	1	1(100.00%)	1	0	0(0.00%)
Total	933	855(91.64%)	681(72.99%)	174(18.65%)	78(8.36%)

*Species not included in the nucleotide MALDI-TOF-MS panel.

### Analysis of nucleotide MALDI-TOF-MS performance for identifying *Mycobacterium* isolates

To evaluate the performance of nucleotide MALDI-TOF-MS in identifying various *Mycobacterium* species, the results of the 933 isolates were compared with those of Sanger sequencing (**Table 2**). Of the 589 *M. intracellulare* samples, the nucleotide MALDI-TOF-MS correctly detected 99.32% (585/589), including 496 *M. intracellulare* and 89 *M. intracellulare* samples mixed with other *Mycobacterium* species. The correct detection rates for *M. abscessus*, *M. kansasii*, *M. avium*, MTBC, *M. gordonae*, and *M. massiliense* were 100% (86/86), 98.46% (64/65), 94.59% (35/37), 100% (34/34), 95.65% (22/23), and 100% (19/19), respectively. However, *M. marseillense*, *M. colombiense*, *M. lentiflavum*, *M. parascrofulaceum*, and *M. interjectum* were not included in the panel and could not be correctly detected using nucleotide MALDI-TOF-MS. Additionally, *M. fortuitum*, *Mycobacterium chelonae*, *Mycobacterium xenopi*, and *Mycobacterium peregrinum* were detected less frequently with an accurate detection rate of 100%. *Mycobacterium scrofulaceum* was detected less frequently, with an accurate detection rate of 0%. Compared to that with Sanger sequencing, the overall correct detection rate of all 933 isolates using nucleotide MALDI-TOF-MS was 91.64% (855/933). Of the 933 isolates, nucleotide MALDI-TOF-MS identified mixed infections in 18.65% of isolates (174/933), and 8.36% (78/933) of the isolates were incorrectly identified as belonging to other species.

We further evaluated the efficacy of nucleotide MALDI-TOF-MS for identifying single-infection *Mycobacterium* isolates, using Sanger sequencing as the reference standard (**Table 3**). The results showed strong agreement between both methods for identifying MTBC, *M. abscessus*, *M. kansasii*, and *M. massiliense* (*k* = 0.907, 0.984, 0.989, and 0.965, respectively). Additionally, nucleotide MALDI-TOF-MS exhibited values greater than 97.00% for the sensitivity, specificity, PPV, NPV, and accuracy for identifying these species, except for MTBC, which was associated with a PPV of 83.33%. A comparison between nucleotide MALDI-TOF-MS and Sanger sequencing for the identification of *M*. *intracellulare*, *M*. *avium*, and *M*. *gordonae* also demonstrated good agreement (*k* = 0.825, 0.776, and 0.878, respectively), with nucleotide MALDI-TOF-MS showed over 90% accuracy for these species.

**Table 3:** The efficacy of nucleotide MALDI-TOF-MS in identifying 759 single-infection isolates compared to Sanger sequencing.

Species*	Sensitivity (%, 95% CI)	Specificity (%, 95% CI)	Predictive value (%, 95% CI)		Accuracy (%, 95% CI)	Kappa value
			PPV	NPV		
*M. tuberculosis* complex	100 (76.14–100.00)	99.60 (98.76–99.92)	83.33 (59.95–94.99)	100 (99.38–100.00)	99.60 (98.79–99.92)	0.907 (0.803–1.000)
*M. intracellulare*	99.20 (97.88–99.77)	79.54 (74.19–84.02)	90.35 (87.57–92.56)	98.10 (95.03–99.43)	92.49 (90.38–94.17)	0.825 (0.782–0.868)
*M. abscessus*	100 (93.68–100.00)	99.71 (98.88–99.99)	97.18 (89.71–99.81)	100 (99.33–100.00)	99.74 (98.98–99.99)	0.984 (0.962–1.000)
*M. kansasii*	98.04 (88.73–100.00)	100.00 (99.35–100.00)	100.00 (91.48–100.00)	99.86 (99.12–100.00)	99.87 (99.18–100.00)	0.989 (0.969–1.000)
*M. avium*	90.00 (68.68–98.43)	98.92 (97.84–99.49)	69.23 (49.85–83.66)	99.73 (98.94–99.99)	98.68 (97.56–99.32)	0.776 (0.641–0.911)
*M. gordonae*	91.67 (61.52–99.79)	99.73 (99.04–99.97)	84.62 (54.55–98.08)	99.87 (99.26–100.00)	99.61 (98.86–99.92)	0.878 (0.807–0.949)
*M. massiliense*	100.00 (74.85–100.00)	99.87 (99.16–100.00)	93.33 (68.16–100.00)	100.00 (99.38–100.00)	99.87 (99.18–100.00)	0.965 (0.896–1.000)

*Agreement of species with low numbers was not quantified using the kappa statistic.

### Analysis of misidentification results via nucleotide MALDI-TOF-MS

Out of the 78 misidentified isolates, which included 10 different species, 69 isolates from these species were not covered in the nucleotide MALDI-TOF-MS panel. These species comprised *M. marseillense*, *M. colombiense*, *M. lentiflavum*, *M. parascrofulaceum*, and *M. interjectum.* In addition, three *M. intracellulare* samples were misidentified as *M. gordonae*, *M. scrofulaceum*, and *M. massiliense*; two *M. avium* samples were misidentified as *M. tuberculosis* complex; one *M. gordonae* sample was misidentified as *M. intracellulare*; one *M. kansasii* sample was misidentified as *Mycobacterium szulgai*; and one *M. scrofulaceum* sample was misidentified as *M. septicum.* Among the 78 misidentified isolates, 33 *M. marseillense* and 17 *M. colombiense* samples from the MAC were incorrectly identified as *M. intracellulare* via nucleotide MALDI-TOF-MS; four *M. colombiense* samples were misidentified as *M. avium*, and one *M. intracellulare* sample was misidentified as *M. avium* (**Table 4**).

**Table 4:** Misidentification results of 78 isolates by nucleotide MALDI-TOF-MS.

Sanger sequencing results	Nucleotide MALDI-TOF-MS results	Number of isolates (n)
M. marseillense*	*M. intracellulare*	33
	*M. abscessus*	1
M. colombiense*	*M. intracellulare*	17
	*M. avium*	4
	*M. mucogenicum*	1
	*M. tuberculosis* complex	1
M. lentiflavum*	*M. avium*	2
	*M. scrofulaceum*	2
	*M. intracellulare*	1
	*M. abscessus*	1
*M. intracellulare*	*M. gordonae*	1
	*M. scrofulaceum*	1
	*M. massiliense*	1
	*M. avium*	1
M. parascrofulaceum*	*M. scrofulaceum*	2
	*M. gordonae*	1
M. interjectum*	*M. intracellulare*	1
	*M. malmoense*	1
	*M. avium*	1
*M. avium*	*M. tuberculosis* complex	2
*M. gordonae*	*M. intracellulare*	1
*M. kansasii*	*M. szulgai*	1
*M. scrofulaceum*	*M. septicum*	1

*Species not included in the nucleotide MALDI-TOF-MS panel.

### Analysis of mixed-infection results via nucleotide MALDI-TOF-MS

Sanger sequencing did not detect mixed infections among the isolates. In contrast, nucleotide MALDI-TOF was able to detect two or more mixed infections associated with 174 isolates. Among these samples, 143 were infected with two species, 29 with three species, and two with four species. Regarding the infection patterns, 44/174 isolates were identified as MTBC mixed with NTM, and the remaining isolates were determined to be mixed infections with different NTM species, with the majority (97/174) showing a pattern of *M. intracellulare* mixed with other NTM species (**Supplementary Table 3**).

## Discussion

Nucleotide MALDI-TOF-MS is a highly effective tool for rapidly detecting nucleic acids, with high sensitivity and throughput (Lee et al., 2016). It is based on multiplex PCR and single-base extension techniques, allowing for direct detection of molecular mass without relying on other signals. Due to the molecular mass differences between different extension products, the target gene can be identified (Wu et al., 2022). This study evaluated the performance of nucleotide MALDI-TOF-MS for identifying *Mycobacterium* species. The results showed that nucleotide MALDI-TOF-MS correctly identified 91.64% (855/933) of the isolates and demonstrated excellent identification capabilities, particularly for *M. intracellulare*, *M. avium*, *M. abscessus*, *M. massiliense, M. kansasii*, and MTBC, which are the most prevalent and significant pathogens associated with human lung diseases (Koh, 2017). The correct detection rate of these species exceeded 94%, and nucleotide MALDI-TOF-MS and Sanger sequencing showed good agreement in identifying these species (*k* > 0.7). Accurate and rapid identification of *Mycobacterium* species is critical for the correct diagnosis and precise treatment of MTB and NTM diseases. Unlike protein MALDI-TOF-MS, which requires complex procedures for protein extraction, nucleotide MALDI-TOF-MS can directly detect DNA extracted from samples, such as bronchoalveolar lavage fluid and sputum, facilitating the direct testing of a wide range of clinical samples (Luo et al., 2018; Rodriguez-Temporal et al., 2018; Li et al., 2022). Additionally, one reaction system can detect more than 20 specifically targeted regions at the same time, and a chip has 384 wells, theoretically allowing for the simultaneous identification of *Mycobacterium* species in 192 samples within 12 hours, from nucleic acid samples to data analysis. These characteristics facilitate rapid and accurate *Mycobacterium* detection in a range of clinical samples.

The nucleotide MALDI-TOF-MS panel was designed to target specific sequence regions characteristic of each *Mycobacterium* species, including MTBC and 30 NTM species. This technology offers more comprehensive detection of *Mycobacterium* species compared to other molecular techniques (Mäkinen et al., 2006; Liu et al., 2012; Xu et al., 2019). As new species are continually discovered, the panel allows the flexibility to add or adjust new species based on clinical needs, which is a key advantage of this technology.

Detecting mixed infections presents a significant challenge for *Mycobacterium* identification. Misdiagnoses of NTM–MTB mixed infections as MDR-TB have been reported in China, and multiple-species NTM infections have been observed in several nations (Shin et al., 2018; Hirabayashi et al., 2019; Huang et al., 2022). Treatment regimens vary substantially across NTM species, particularly for the MAC, the *M. abscessus* complex, and *M. kansasii* (Daley et al., 2020). Therefore, the reliable distinction of *Mycobacterium* species from mixed infections is critical. Currently, there is no gold standard method to detect and identify multiple NTM infections (Khieu et al., 2021). Sanger sequencing can only detect a relatively high proportion of species in isolates and has limited ability to detect mixed infections (Liang et al., 2020). During the detection process of nucleotide MALDI-TOF-MS, the molecular masses of different single-base extension products vary, allowing for the differentiation of mixed infections by checking whether the detection peaks appear at their respective molecular mass positions. Furthermore, the mixing of target genes from different species causes no interference between different peaks, thus enabling the differentiation of mixed infections. In this study, nucleotide MALDI-TOF detected 44 isolates as MTBC mixed with NTM and the remaining isolates as mixed infections with different NTM species. Sanger sequencing only detected a single species. Among these mixed infections, the possibility of contamination of sputum samples or transient colonization cannot be excluded, especially with less clinically relevant species such as *M. gordonae* and *M. mucogenicum* (Eckburg et al., 2000; Moiz et al,. 2020).

In this study, there were misidentification results in 78 isolates by nucleotide MALDI-TOF, which were inconsistent with the results of Sanger sequencing. The following are possible reasons for the inconsistency between the results of these two methods: First, species outside the panel coverage, such as *M. lentiflavum*, *M. parascrofulaceum*, and *M. interjectum*, could not be correctly identified and require additional detection methods. Therefore, the nucleotide MALDI-TOF-MS panel should be improved to expand the scope of species identification and accommodate regional variation in species distribution. Second, Sanger sequencing identified species by detecting conserved genes (16S-23S rRNA gene internal transcribed spacer, *rpoB*, and *hsp65*). The analysis of the results was dependent on public databases, and the lack of insight into sequence quality and contemporaneity in public databases raised the possibility of misidentification (Joao et al., 2014; Forbes et al., 2018). Third, the design of nucleotide MALDI-TOF-MS primers was primarily based on the insertion sequence or characterization sequence regions of each species. Differences in primer design between the two methods may also account for the inconsistent results. Fourth, our results demonstrated the limited ability of nucleotide MALDI-TOF-MS to distinguish between species within the same complex or group, particularly the MAC. *Mycobacterium avium*, *M. colombiense*, *M. marseillense*, and *M. intracellulare* are part of the MAC family and are closely related or exhibit cross-reactivity in specific regions (Boyle et al., 2015). Nucleotide MALDI-TOF-MS could not accurately identify these species at the species level. Similarly, several species within the MAC and MABC cannot be differentiated using protein MALDI-TOF MS or multicolor melting curve analysis (Luo et al., 2018; Xu et al., 2019). Currently, there are no significant differences in treatment principles between species within the MAC (Daley et al., 2020). The accuracy of identification results is determined by the selection of species-specific loci and the design of primers, and the specificity of primers should be optimized in the future.

The current study had some limitations. The nucleotide MALDI-TOF-MS system used in this study had two wells to detect MTBC and 30 NTM species. However, our study did not include *Mycobacterium marinum*, *M. chimaera*, *Mycobacterium simiae*, *Mycobacterium smegmatis*, or other species beyond the scope of the nucleotide MALDI-TOF-MS panel. Therefore, the ability to identify these species could not be evaluated. Future studies should include reference standards and clinical isolates of other species. Second, although this study included a large number of clinical isolates, the limited number of samples of some species, including *M. fortuitum*, *M. chelonae*, *M. xenopi*, *M. scrofulaceum*, and *M. peregrinum*, resulted in unrepresentative data. Third, mixed-infection isolates detected via nucleotide MALDI-TOF-MS were not confirmed using any other method because of the limited conditions. To confirm the mixed-infection isolate results in future studies, methods that can identify mixed infections should be used. Fourth, various clinical samples, not just isolates, should be selected to confirm the accuracy of the *Mycobacterium* identification results of nucleotide MALDI-TOF-MS. In summary, our study confirmed the accuracy of nucleotide MALDI-TOF-MS for identifying different *Mycobacterium* species and its performance in detecting mixed infections. This could thus be a promising approach for identifying MTBC and the most common clinical NTM species.

## Data availability statement

The original contributions presented in the study are included in the article/**Supplementary Material**. further inquiries can be directed to the corresponding author.

## Author contributions

YZ: Data curation, Writing – original draft. ZL: Funding acquisition, Methodology, Writing – review & editing. LP: Data curation, Methodology, Writing – review & editing. BL: Data curation, Methodology, Software, Writing – review & editing. KW: Data curation, Validation, Writing – review & editing. MZ: Visualization, Writing – review & editing. XW: Methodology, Writing – review & editing. JP: Methodology, Resources, Writing – review & editing.

## Supplementary material

Supplementary Table 3 The 78 results highlighted in red refer to the misidentification results of 78 isolates by nucleotide MALDI-TOF-MS. The 174 results highlighted in green refer to the mixed-infection results via nucleotide MALDI-TOF-MS.
