# Supplementary material for: Evaluation of nucleotide MALDI-TOF-MS for the identification of Mycobacterium species
Source: Front Cell Infect Microbiol. 2024 Feb 6;14:1335104. doi: 10.3389/fcimb.2024.1335104 (PMC10876993; doi:10.3389/fcimb.2024.1335104)
Supplement: Supplementary file 1 [file Table_1.doc]

Supplementary Table 1. The information of extension reaction designed for *Mycobacterium* identification

| Well | ID | Target | Molecular mass  of extension primer | Extension Base | Molecular mass  of extension product |
| --- | --- | --- | --- | --- | --- |
| Well1 | M.abs | *M. abscessus* | 7951.2 | A | 8278.3 |
| Well1 | M.asi | *M. asiaticum* | 7321.8 | G | 7609 |
| Well1 | M.che | *M. chelonae* | 8564.6 | C | 8811.7 |
| Well2 | M.chim_2 | *M. chimaera* | 6695.4 | A | 7022.5 |
| Well1 | M.for | *M. fortuitum* | 7298.7 | G | 7545.9 |
| Well2 | M.gast_1 | *M. gastri* | 4544 | T | 4871.1 |
| Well2 | M.gena_2 | *M. genavense* | 6119 | G | 6366.2 |
| Well2 | M.gilv_1 | *M. gilvum* | 7351.8 | T | 7623 |
| Well2 | M.gor_1 | *M. gordonae* | 5554.6 | G | 5841.8 |
| Well1 | M.hae | *M. haemophilum* | 8340.4 | G | 8627.6 |
| Well1 | M.int_n | *M. intracellulare* | 4625 | C | 4872.2 |
| Well1 | M.kan | *M. kansasii* | 6711.4 | T | 7038.5 |
| Well1 | M.mal_n | *M. malmoense* | 7305.7 | C | 7592.9 |
| Well2 | M.mar_erp_n | *M. marinum* | 5164.4 | C | 5411.6 |
| Well2 | M.mar_IS2404_n | *M. marinum* | 6485.2 | A | 6756.4 |
| Well2 | M.mass_1 | *M. massiliense* | 7992.2 | C | 8239.4 |
| Well2 | M.muco_2 | *M. mucogenicum* | 5727.7 | G | 5974.9 |
| Well2 | M.pere_2 | *M. peregrinum* | 5498.6 | C | 5785.8 |
| Well2 | M.phle_1 | *M. phlei* | 6151 | C | 6398.2 |
| Well1 | M.scr | *M. scrofulaceum* | 4811.1 | A | 5138.2 |
| Well2 | M.sept_1 | *M. septicum* | 6536.2 | C | 6783.4 |
| Well1 | M.shi_n | *M. shimoidei* | 5309.4 | T | 5636.5 |
| Well2 | M.sim_2 | *M. simiae* | 5148.4 | G | 5395.6 |
| Well1 | M.sme | *M. smegmatis* | 6179 | C | 6426.2 |
| Well1 | M.szu | *M. szulgai* | 5186.4 | G | 5433.6 |
| Well2 | M.terr_2 | *M. terrae* | 5431.6 | A | 5758.7 |
| Well2 | M.triv_1 | *M. triviale* | 4577 | T | 4904.1 |
| Well1 | M.ulc | *M. ulcerans* | 7417.8 | A | 7744.9 |
| Well1 | M.xen | *M. xenopi* | 7994.2 | C | 8241.4 |
| Well1 | M.avc_2 | *M.avium* | 6486.2 | A | 6813.3 |
| Well1 | M.avc_1 | *M.avium* | 6750.4 | C | 6997.6 |
| Well1 | M.cel | *M.celatum* | 4984.2 | G | 5271.4 |
| Well1 | MTBC | *Mycobacterium tuberculosis* complex | 5589.6 | C | 5876.8 |
| Well1 | MTBC_IS1081 | *Mycobacterium tuberculosis* complex | 5698.7 | A | 6025.8 |
